# The European Union and Public Health Emergencies: Expert Opinions on the Management of the First Wave of the COVID-19 Pandemic and Suggestions for Future Emergencies

**DOI:** 10.3389/fpubh.2021.698995

**Published:** 2021-08-20

**Authors:** Marie Gontariuk, Thomas Krafft, Cassandra Rehbock, David Townend, Loth Van der Auwermeulen, Eva Pilot

**Affiliations:** ^1^Department of Health, Ethics and Society, Faculty of Health, Medicine and Life Sciences, Care and Public Health Research Institute, Maastricht University, Maastricht, Netherlands; ^2^Faculty of Law, Centre for Government and Law, Hasselt University, Hasselt, Belgium

**Keywords:** COVID-19 pandemic, European public health, health emergencies, health policy, European governance, international health regulations, public health emergencies of international concern, joint procurement mechanism

## Abstract

**Objective:** The first wave of the coronavirus SARS-COV-2 pandemic has revealed a fragmented governance within the European Union (EU) to tackle public health emergencies. This qualitative study aims: 1) to understand the current EU position within the field of public health emergencies taking the case of the COVID-19 as an example by comparing and contrasting experiences from EU institutions and experts from various EU Member States at the beginning of the pandemic; and, 2) to identify and to formulate future EU pandemic strategies and actions based on experts' opinions.

**Methods:** Eighteen semi-structured interviews were conducted with public health experts from various European Member States and European Commission officials from May 2020 until August 2020. The transcripts were analyzed by Thematic Content Analysis (TCA), mainly a manifest content analysis.

**Results:** This study demonstrated that the limited EU mandate in health hinders proper actions to prevent and tackle infectious disease outbreaks, such as the COVID-19 pandemic. The results showed that this limitation significantly impacted the ECDC, as the Member States' competence did not allow the agency to have more capacity. The European Commission has fulfilled its role of coordinating and supporting the Member States by facilitating networks and information exchange. However, EU intra- and inter-communication need further improvement. Although diverse EU instruments and mechanisms were found valid, their implementation needed to be faster and more efficient. The results pointed out that underlying political challenges in EU decision-making regarding health emergencies hinder the aligned response. It was stated that the Member States were not prepared, and due to the restriction of their mandate, EU institutions could not enforce binding guidelines. Additionally, the study explored future EU pandemic strategies and actions. Both, EU institutions and national experts suggested similar and clear recommendations regarding the ECDC, the investment, and future harmonized preparedness tools.

**Conclusion:** The complex politics of public health at the EU level have led to the fragmentation of its governance for effective pandemic responses. This ongoing pandemic has shed light on the fragility of the political and structural systems in Europe in public health emergencies. Health should be of high importance in the political agenda, and robust health reforms at the local, regional, national, and EU levels are highly recommended.

## Introduction

Public health emergencies have been a part of the global policy agenda for more than two decades. Their international recognition and potential impact on societies worldwide caused by the spread of infectious diseases make them critical and highly influential phenomena globally. Disease outbreaks, such as the severe acute respiratory syndrome (SARS) and Influenza (H1N1), influenced the World Health Organization (WHO) to reform the International Health Regulations (IHR) (1), and caused national public health agencies to set up and invest in health emergency plans at the beginning of this millennium (2). However, the novel coronavirus SARS-COV-2 from December 2019, better known as the COVID-19 pandemic, exposes shortcomings in outbreak preparedness and response at the global level (3, 4).

The European Union is one of the most affected areas, declaring a state of emergency in early March 2020 (5). By engaging its 27 European Union Member States (EU MS), the European Union (EU) was expected to generate immediate, comprehensive, and harmonized responses and collective actions within the scope of its treaty-based competence at the beginning of the crisis (6). Limiting the spread of the virus, providing medical equipment, promoting research, and the importance of strengthening solidarity, cooperation, and exchange of information between the EU MS were highlighted as priorities on the 10th of March 2020 by the European Council (7).

Based on the Treaty on the Functioning of the EU (TFEU), EU institutions have limited powers to take action in the public health field. They may only “support, coordinate or supplement” EU MS, and only a few binding public health policies are enforced at the EU level [TFEU, Article 168 (1, 8)]. This implies that the legally binding acts of the Union adopted under the provisions of the Treaties on public health cannot involve any harmonization of the laws or regulations of the Member States. Concerning outbreaks, the European Parliament and Council Decision on Serious Cross-border Threats to Health (No 1082/2013/EU) empower the EU institutions to have a complementary role in coordinating the EU MS' actions. The role of the European Center for Disease Prevention and Control (ECDC) according to the legislation is to perform risk assessments and surveillance and provide the EU MS with guidelines and recommendations. In terms of crisis management, the European Commission (EC) has more responsibility than the ECDC. Notably, the Health Security Committee (HSC), as part of the Directorate-General for Health and Food Safety (DG SANTE), is accountable for coordinating responses during public health crises among EU MS (9).

Furthermore, the EU strengthened its disaster risk management elements by upgrading the Civil Protection Mechanism in 2019. The latest enhancement, the RescEU, is anchored at Directorate-General for European Civil Protection and Humanitarian Aid Operations (DG ECHO) (10). Under EU MS competence, the mechanism is operational for coordinating and strengthening EU MS disaster relief capacities during a crisis and disaster preparedness and training. For strengthening preparedness, another EU tool, the Joint Procurement Agreement, came into force in 2014. Thereby, EU MS voluntarily joined a procedure to purchase medical equipment and products, especially vaccines, collectively; the EC is in charge of managing this procedure (6, 9).

Yet, the EU's capacity to deploy those instruments and tools has been seriously challenged during the COVID-19 crisis (11). Additionally, criticism of fragmented governance and a disunited EU has arisen as the EU MS have adopted divergent strategies to monitor and contain the outbreak (7, 12): Germany, for example, focused at the beginning on a strategy of regular and extensive testing of its citizens; Italy, Spain, and Belgium introduced a strict quarantine-regime with restricted displacements; Sweden and The Netherlands opted for voluntary quarantine and social distancing measures (13, 14).

This fragmented governance within the EU have impacted the EU response and actions for tackling the pandemic. Thus, it is essential to assess what has been in place and what would be needed to cope with future health crises in the European Union. For this, internal and external insights are necessary. Hence, this study has a two-fold aim: 1) to understand the current EU position within the field of public health emergencies taking the case of the COVID-19 as an example by comparing and contrasting experiences from EU institutions and experts from various EU MS at the beginning of the pandemic; and, 2) to identify and to formulate future EU pandemic strategies and actions based on experts' opinions.

## Materials and Methods

### Design of the Study

Using the initial phase of COVID-19 pandemic as a case in point, a qualitative study design was applied to explore diverse perspectives on the EU's position regarding public health emergencies and its potential future actions.

### Participants and Recruitment

A purposive sample method was adopted to approach the participants. The participants were selected based on their involvement in the EU and their expertise in public health emergencies. On the one hand, European officials from different EU institutions dealing with public health emergencies were contacted to collect EU internal opinions and experiences. On the other hand, public health officials from EU MS Health Ministries and national institutions, and public health experts from all EU MS were contacted to gather external opinions and experiences. Participants will be referred to in the results according to a given participant code (see Table 1). Participants were recruited through social media (LinkedIn) and direct contact via email.

**Table 1:** Study participants characteristics.

**Participant code**	**Functions**
P1	Senior Commission Official, ECDC
P2	Expert in EU Global Health, Finland
P3	Advisor, Health and International Relations, FPS Public Health, Belgium
P4	Commission Official, DG DEVCO
P5	Public health doctor and Epidemiologist, Instituto Nacional de Saúde, Portugal
P6	Senior Member of Committees at the Ministry of Health, Lithuania
P7	Public health consultant, Ministry of Health, Malta
P8	Senior Commission Official, DG SANTE
P9	National Civil Protection Expert, Luxembourg
P10	Professor, National Institute of Public Health, Croatia
P11	High-level specialist, Department of Health Security, Finnish Institute for Health and Welfare (THL), Finland
P12	Senior Public health expert and Consultant, France
P13	Expert in Global Health Security, UK
P14	Senior Commission Official, DG ECHO
P15	Counsellor, Ministry of Foreign Affairs, European Union and Cooperation, Spain
P16	Epidemiologist and Infectious disease expert, Istituto Superiore di Sanita', Italy
P17	Senior Advisor, Health, Demographic Change & Wellbeing, The Netherlands
P18	Medical doctor, Federal Ministry of Social Affairs, Health, Care and Consumer Protection, Austria

### Data Collection

Eighteen experts were interviewed from May 2020 until August 2020: 17 online semi-structured interviews were conducted; one participant submitted the answers to the questions in written form due to COVID-19 related time constraints. The interviews were conducted in English via Skype, Zoom, or phone calls. The interview lasted around 30 min (range: 20–65 min), depending on the participants' availability. The interviews were audio-recorded and then transcribed. One participant did not consent to be audio-recorded or quoted, but the participant agreed to the interview's content derived from the interviewer's notes.

The semi-structured interview guide (see Supplementary Materials) entailed open-ended thematic questions regarding the experiences and insights about public health crises in general, on the current COVID-19 pandemic, and the EU. This allowed us to obtain diverse opinions on the current situation and suggestions for future health crises from the participants' perspective.

### Data Analysis

The transcripts were analyzed by Thematic Content Analysis (TCA), mainly a manifest content analysis, using the qualitative analysis software ATLAS.ti (ATLAS.ti Scientific Software Development GmbH, Berlin, Germany https://atlasti.com/). The transcripts were read and analyzed multiple times to identify overarching themes while combining coding units from each transcript (15). The following themes were identified: 1) Experiences from EU institutions during the COVID-19 crisis; 2) Experiences from some EU MS during the COVID-19 crisis; 3) Suggestions for future actions for public health emergencies at the EU level. Sub-themes are further illustrated in the results section.

### Ethical Consideration

The study received ethical clearance (FHML/GH_2018.076) from Maastricht University, The Netherlands. All participants signed an informed consent form and gave their consent orally at the start of the interview.

## Results

A total of 18 participants were interviewed. Four were from the EU institutions, twelve were representing EU MS, and two were experts on global health security at the EU level. Table 1 presents the participant codes used throughout the results to refer to the participants and their respective functions.

The result section is structured into three overarching themes:

(1) EU Institution's perspectives on the Covid-19 pandemic;

(2) EU MS experts' experiences on Covid-19 in the European context;

(3) EU Institutions' and EU MS experts' suggestions to enhance future pandemic preparedness and response.

### COVID-19 Crisis: The European Union Institutions' Perspective

#### Internal EU Collaboration

All interviewees gave varied insights into the EU institutions' internal collaboration processes, highlighting aspects of communication and cooperation. Directorate-General for Health and Food Safety (DG SANTE), the Directorate-General for International Partnerships (DG DEVCO) and the Directorate-General for European Civil Protection and Humanitarian Aid Operations (DG ECHO) representatives all mentioned that their directorates collaborated well by cooperating and communicating daily during the crisis (P4, P8, P14). The ECDC participant (P1) stated:

“So having an agency in Europe that is a real public health agency will probably increase the capacity to interact with other sectors, you don't have this capacity, we have to move very, very, very [cautiously] through a maze of contacts and cooperation to cooperate with other sectors. We do this, of course, but it's complicated. We don't have a direct link to any sectors. And it's obvious that public health is not just health, it's much more than that” (P1)

It is rather complicated for them to directly collaborate and cooperate with other sectors within and outside the EU institutions.

#### Coordinating the Joint Procurement

Two participants mentioned Joint Procurement as part of DG SANTE's coordinating role during a health crisis (P8, P14). The crisis unit of DG SANTE can organize Joint Procurement for medical countermeasures (P8). During this crisis, DG SANTE managed several procurements as requested by the EU MS, such as protective equipment, ventilators, laboratory equipment, and a tender for ICU medicines (P8). Positively, DG SANTE was active in coordinating the needs of the MS in terms of equipment:

“One thing which we have seen has been this joint procurement-related issues where the EU and especially the DG SANTE has been very active in coordinating any need that the member states have in set up again with the personal protective equipment with the ventilators, so they have assisted the member states to come up and build up the critical mass for the purchases by combining all member states requests, and that's where the DG SANTE has played a key role” (P14)

#### Challenges

Various challenges, mainly related to EU MS and EU bureaucracy, were addressed by all participants. In general, the interaction between EU MS and EC in the field of public health is complex. EU MS are very conservative concerning their health mandate, and this results in diverse opinions causing conflict in decision-making at the EU level for public health (P4). Related to the COVID-19 pandemic, EU MS acting on its own was mentioned several times as a challenge for the EU institutions.

First, as observed and experienced in the past, solidarity and global health principles during a public health crisis are often of minor importance for national states. When an emergency strikes, it affects all states at the same time. Thus, each state's main priority is to manage its crisis at its national level (P1). Moreover, a participant explained that the Civil Protection Mechanism relies on the solidarity of the MS and the challenge was that the COVID-19 crisis happened simultaneously in all MS, lowering the cooperation and thus solidarity among them (P14). Joint Procurement for a COVID-19 vaccine was thus also not perceived as an expression of real solidarity among EU MS. As one participant explained the EU MS releasing vaccines first, would produce it first for their respective population and then for the other EU MS (P1).

Second, a participant felt that the system involved in the cooperation among EU MS has been under particular stress as EU MS resources are principally devoted to dealing with the outbreak at their national level. Therefore, coordination with other EU MS using EU level mechanisms was sometimes seen as a secondary priority (P8).

“I think the main problems are not at the EU level but the problems with national level. So I don't know if increasing the competence of the European Union or the coordination mechanisms is going to solve the problem if the problem is lack of resources or capacity at the national level […] The real problem is the shortage of manpower, preparedness, lack of capacity at the national level, I don't see what adding a layer of coordination ever will do.” (P8)

A third challenge perceived by DG SANTE and DG ECHO representatives was about crisis preparedness at the EU MS level (P8, P14). The participant from DG SANTE perceived a lack of transparency regarding the state of preparedness reported by certain EU MS, which could have led to a lack of trust between MS. The lack of capacity and resources at the national level was considered problematic and cannot be solved solely by the EU coordination mechanism. The preparedness plans are developed, and the challenge is for EU MS to fully implement them (P8). Furthermore, the DG ECHO participant explained that some EU MS did not want to share preparedness information with EU institutions as they considered this to be classified data, complicating the cooperation between the EU and EU MS (P14).

“But the biggest challenge is that the MS are responsible for their health systems. They know what they're investing in, you know what's happening. And it's difficult at a central level and the European Union to know, in detail, about what the state plays at the national level. This is additionally complicated by the fact that many MS have regional levels. […] And to make sure that there's transparency because if you imagine that your neighbor's house is in good condition and then suddenly collapses into your garden, on top of your kids, I mean, you're not going to be very happy. And that's really what we've discovered here that some MS have been not fully transparent with themselves and with others, and that leads to a lack of trust between MS” (P8)

Lastly, underfunding of public health and public health emergencies was repeatedly mentioned as an issue. The attractiveness of investment in public health, preparedness and development aid for health is at a low level politically at the EU and MS levels as the economic prosperity of health is not recognized (P1, P8, P4), as stated by P1: “Sometimes public health is not considered as an investment because there's no real, tangible economic return. Although it is very difficult because the health of the citizens is the basis of the economy.”

### COVID-19 Crisis: Experiences From Some EU Member States

#### EU-Level Coordination and Communication

The majority of the participants mentioned that one of the strengths of the EU institutions during the pandemic was its coordinating role with the EU MS, in particular, the network established and the exchange of information at all levels (P3, P9-P11, P13, P15-P18):

“The EU is a forum where you can come together and exchange and I think that was fully useful at all levels from the highest level down to working groups. […] And everybody worked, there was a lot of exchange, which is often also either triggered by the EU or by Member State, I think on expert level there is even an informal exchange. That's really a strength of the EU […] When you have a platform where you can come together and exchange. And the next step is to coordinate together, and EU tried to propose in certain domains, like the repatriating flights for people from all over the world […] And up until now it worked very well” (P9)

However, a few participants argued that the coordination mechanism from the EU was too slow as it underestimated the importance of the virus, or that the EC does not have enough legislative power to coordinate public health reactions thoroughly. As a result, some EU MS felt the need to act on their own since the onset of the pandemic (P2, P3 P7, P10, P12, P15):

“Well, the truth is I think that each country really went on its own. Basically, there was a very weak EU response initially sadly, EU wide response. There was very little coordination at the EU level and each country felt the need to respond to its own circumstances.” (P7)

Further, although there was a lot of interaction and communication between the ministries and agencies, one participant criticized the internal coordination and communication between the different EU directorates. Instead of interacting with each other, the DGs were viewed as working in silos. Some EU MS received duplicated information from different EU DGs, which confused them about the EU coordination organization (P11):

“But I have noticed that when it comes to the EC, DG SANTE, DG ECHO, MOVE and all these different agencies, I think there could be room for improvement in terms of the interaction between those as well. I think they may work in silos instead of really talking to each other. And then there are a number of mechanisms now in the EU level, activated response, mergers, response mechanisms, and sometimes, I get emails from all kinds of instances and maybe too much, maybe sometimes you feel like okay, there's this and that and then there and you really get the idea that who is actually coordinating what” (P11)

On the international level, two participants mentioned that the EU took a step forward as a public health actor in this crisis by proposing a resolution at the World Health Assembly for requesting affordable and accessible COVID-19 vaccines, which, in their opinion, strengthened the EU position during this pandemic at the global level (P2, P3):

“The EU did a very good job, I think at the level of WHO. There the EU proposed a resolution last week at the World Health Assembly, which mainly states that immunization should be considered global public good for health and that everyone should have access to affordable vaccines. That was during the WHA that the EU negotiated the resolution at the global level, the EU did a good job toward other countries.” (P3)

#### Joint Procurement

The EC's Joint Procurement mechanism was recognized as a great initiative of the EU in this crisis, as the EU MS could not manage to obtain protective equipment at their national level at the beginning of the crisis (P2, P7, P10, P12, P15). However, one major criticism was that the mechanism could have been launched earlier and faster to avoid any equipment waste (P2, P7, P15):

“The commission with actions like joint procurement, they don't really release very quickly the joint procurement, but now 3 months, 4 months later, the results are very small and so, every member state has already solved the problem by themselves […] I think the joint procurement is very clear but there were problem of personal equipment, problem of the Spanish government, the French government, the German government, not the problem of the Commission” (P15)

#### The European Center for Disease Prevention and Control

Most of the participants mentioned the ECDC's role, but the opinions on the effectiveness of its function were conflicting. Overall, the ECDC had performed well within the scope of its mandate in terms of early warning, data analyses, information exchange, and recommendations (P2, P3, P5, P6, P9, P11, P12, P16, P18). Two participants stated that the ECDC was active and had been collaborating with them since the beginning of the pandemic (P6, P16):

“We had early TCs (teleconferences) with them, the ECDC, and then they came on, we had a delegation from the ECDC come to Italy, discuss together what to do in the middle of the emergency. And we do have connections with them through webinars, we are in contact with them for surveillance and a number of other activities […] they were there from the beginning. They've always been there. […] I think what was really helpful is that they showed a lot of flexibility in the way they were accepting and interacting with the member states […]” (P16)

Only one participant highly criticized the ECDC, which, in his opinion, is a “*very, very much political type of organization with very little public health or evidence-based policies drive”* (P7) and added that ECDC's initial response was feeble and ineffective. As a result, EU MS had to handle the pandemic by themselves (P7).

Although most participants were positive about the ECDC's actions during the pandemic, several negative comments were made. The majority criticized its mandate, and suggested that some improvements in that regard should be considered. Further, the ECDC would not have enough capacity in terms of staff and responsibilities, hampering a strong leadership in public health emergencies, which entails providing strong standardized guidelines for EU MS (P7, P9-P12, P15). And finally, the majority claimed that the ECDC was not visible enough to the public and the EU MS (P2, P3, P7, P9-P11):

“From the ECDC side, they actually published the recommendations, they're available on the website. And I imagine that there's some high-level meetings where they interact, but from the public's point of view, there was really not much interaction or intervention from that side” (P5)

#### Public Health Emergency Preparedness at the EU Level

The actual level of preparedness for public health crises in the EU, especially outbreaks and pandemics, was highly criticized by most participants. During the past years, there was a lack of interest regarding health in general at the EU MS and the EC, neglecting contagious diseases in the European region. Health system strengthening and efficiency was perceived as the primary focus (P2, P3, P10- P13, P15 -P18):

“There was a very big panic reaction among many member states and that was also driven by insufficient preparedness at the level of the EU for health crises. […] I get the impression that we somehow assume that this crisis wasn't avoidable and that it just happened and that you can only deal with it. But I think that a lot would have been avoidable with better preparedness and coordination” (P3)

Additionally, some participants emphasized that the EU, including its MS, allocated most of the global health security funds to developing countries and underestimated the budget for public health crises in its region, impeding on both EU and national outbreak management (P2, P6, P11, P13, P16, P17):

“I think there's been a lot of work done by the ECDC and the MS. But, it's all about resources. We have been talking about pandemic preparedness for years when it comes to global health security and how countries should be assessed, how countries should help each other […] There's a lot of money and resources we use for military threats but there's not much we use actually for biological threats. I mean, the preparedness and against biological threats. So, I think there's been work but obviously it's heavily under resourced, underfunded.” (P11)

Moreover, the EU and EU MS have based their actions against the COVID-19 pandemic on the H1N1 plans at the onset of the pandemic, which according to the experts was not appropriate (P2, P12, P16). In relation to this, one participant strongly criticized the non-implementation and deficiencies in considering the International Health Regulations (IHR) by the MS (P12):

“I must say that the International Health Regulations started to be less and less concerned for most countries in the world […] And now we are saying “What should we do to be better prepared?,” and I say, you don't need to think long for it. We have IHR, change it a bit if needed but we have the tool. It's just an issue with the governance of our Member States, and especially in Europe” (P12)

#### Political Barriers

One of the rationales behind the EU's poor leadership that was stated by some participants during the current pandemic is its institutional complexity. The EU has multiple political levels of decision-making and a lot of bureaucracy, impacting the speed of its responses and actions. Furthermore, the EC's actions are restricted, especially in public health, by the Council's and MS decisions (P3, P7, P10, P11, P15, P17).

“There wasn't strong leadership from the European Union. […] European Commission as a governing institution, you have a lot of bureaucracy” (P10)

### Future Public Health Emergencies Actions at the EU Level

#### Suggestions for Improvement From the Perspective of EU Institutions

The main suggestion was to propose a revision of specific EU instruments and institutions, such as the European Parliament and Council Decision on Serious Cross-border Threats to Health (No 1082/2013/EU) and the ECDC mandate, but without necessarily going as far as suggesting a Treaty change. The majority of the participants emphasized developing a process of frequent audits to help the EU MS with their preparedness level, managed by the ECDC (P1, P8, P14):

“I think the problem is not the plan. The problem is that it has to be implemented. And this is why we're examining at the moment the possibility of computing visits and audits with 6-months reports to the European Council so that the transparency of the whole operation becomes open. And that there is a public examination and transparency on what we found in these visits. If we discover that the hospitals have no staff or they have no equipment or there are no stockpiles, or that there's no provision made for infection control, or whatever the issue might be, the fact that these figures are publicly reported to the Council every 6 months will really, I think, drive an improvement.”(P8)

Additionally, the flow of information and communication between the different EU institutions and internal sectors within the institutions (i.e., DG SANTE and DG ECHO) should be improved and facilitated in order to respond faster (P1, P4). One participant recommended allocating more funds to global public health security at the EU level to ensure better preparedness into the future (P4). Furthermore, one participant suggested increasing EC capacity for managing and investing in stockpiling emergency equipment at the EU level in order to avoid any shortage in the future (P14). Lastly, the EC should perform a case study of the current pandemic to identify shortcomings and to overcome them in the future (P8, P14):

“In the future, we hope that we as the Commission would be in a position to purchase equipment to tackle, and I mean to purchase and own and stock equipment for the future that we could then in the case that all the member states are hit at the same time, there would be an EU reserve, that could then be used for assets, in order to assist the member states. That's what we're trying to do, our current legislation does not allow us to do it fully, I mean, independently, we always have to go through a member state who would be what we call a hosting hub for us and they would do the purchase, we will finance it, but they will do all the practical arrangements and they will also stock those goods. So basically, now we would like to move one step further and have our own let's call them EU stockpiles […] and building our own, let's say EU resilience” (P14)

#### Suggestions for Improvement From the Perspective of EU Member States Public Health Experts

The experts had several suggestions for improving the future of public health emergencies responses at the EU level. First, the mandate of the ECDC should be revisited. It should have a more substantial role in crisis coordination, more responsibility in terms of assessing preparedness at the national level, and it should receive more resources, both in terms of finance and personnel (P3, P5, P7, P9, P11, P15, P18):

“I think the position of ECDC should be reinforced. It should be possible that they give clearer and more binding advice to individual MS, that they have more visibility and a stronger role in crisis coordination. And still within the scientific role but nevertheless a stronger position” (P3)

And:

“ECDC probably would need to get resources in order to, for one, capacitate the response within the different countries. So, they need to be much more proactive in trading the resources probably would need to develop these sort of rapid response teams at the European level that can be easily mobilized and quickly mobilized to respond to the different settings” (P5)

Second, some experts recommended that EU competence should be increased in relation to crisis management. The EU should improve the coordination at all institutional levels, internally and externally, and clearly define every actor's tasks for managing a crisis. Concerning this, in the future the EU should have a leading role in public health crisis management as it appeared to have not been unified in this pandemic (P3, P7, P9-P11, P15-P17):

“I think that institutional arrangements and leadership is key to deal with a crisis. […] and in my view there have been a lot of attention to technical capacities in countries like having a strategic stock for PPE and strategic stocks for medicine and it is very important to have sufficient hospitals and beds and nurses and so on, but the political leadership and the institutional context to allow for good crisis coordination is equally important but received insufficient attention, and this should change both at Belgium level and the EU level.” (P3)

Additionally, the EU should develop a set of standardized guidelines and/or strengthen the Joint Preparedness planning (such as the IHR), which all EU MS should follow in the future to avoid incoherence and confusion (P3, P5, P9-P11, P16, 18). Two participants emphasized that the EU should facilitate standardized *training* rather than standardized *guidelines* to respond effectively in the next pandemic (P6, P16):

“I think that it's great to have the plan on paper. But once you have it on paper, we have to make simulations and try to see how this could come together. And we can't expect it to come together naturally during an emergency. That's not going to happen if we want to have some kind of international consensus some kind of way forward, which is synced. Then, the more we exercise, the better” (P16)

Third, although they acknowledged the process-related difficulty, some experts recommended having more substantial and better health policy at the EU level (P2, P3, P5, P12, P17). Two experts suggested strengthening the content and application of the European Parliament and Council Decision on Serious Cross-border Threats to Health (No 1082/2013/EU) to be better prepared for future health crises and facilitate contact tracing in the whole region without the need to close national borders (P3, P17). Moreover, another expert highlighted the need to change and adapt the IHR, and suggested that the EU should be stricter regarding its implementation (P12):

“International health regulation is a key issue, but we need to change it, then to adapt it and the EU but not only should be more pushy on following implementation, evaluating on auditing what's being done in countries” (P12)

Next, the EU, including its EU MS, should reconsider their allocation of budgets to the public health sector and increasing that funding (P7, P11, P13, P15, P16, P18). One participant highlights the need to invest in global health security to prevent future pandemics (P11). Another participant suggested developing a financial tool at the EU level that could increase the capacity to react quickly (P15).

“So obviously, I think the EU and the Commission needs to respond also by providing funding for public health initiatives public health research even more than it used to be before and also provide adequate structures within the commission to be able to respond to the needs of different countries and member states from a health perspective”(P7)

And

“Investing in global health is very important because if we want to stop pandemics […] We have to find the root cause and understand where it all comes from. […] I do think the EU specifically should play a strong role in global health. […] EU should step in and help out because it's not only again, I just want to emphasize that it's not only for helping the countries in the south, but it's also benefiting us because we can then stop the threats at their sources” (P11)

Finally, experts suggested to have a stockpiling of protective equipment for future public health emergencies at the EU level (P9, P15, P7), or to ensure the production of those in the European region (P12):

“We have faced many difficulties with personal equipment, with ventilators, with medicines, because the virus happened in different member states. So, we need to react very quickly. So, we need to learn for the future that is not enough with role preparation, we need to be prepared, having a stockpile, more coordinated approach in different situations […] We need some stockpiling. We need to create rescue to provide essential medical devices, personal equipment, essential medicines, stocks of ventilators, etc.” (P15)

#### COVID-19: Opportunities for Changes in Public Health Emergency at EU Level

According to most participants, the COVID-19 pandemic is an opportunity for positive changes in public health emergencies at both EU and EU MS levels (P2, P3, P6-P10, P14, P16-P18). The experts foresee a strengthened EU role in crisis management and a more robust public health community in general after this health crisis. However, some experts were more skeptical and doubtful about the future initiatives' longevity considering the past outbreaks and the short-term nature of the lessons learned. The importance of public health, once this crisis is over, maybe overshadowed by market-based priorities (P1, P2, P5, P7, P11, P12):

“I would like to think that, given what happened, that there will be more resources, more funding toward public health, but unfortunately, I don't believe it will actually happen. I think that once this sort of gets sorted out, we'll just go back to business as usual in the usual priorities” (P5)

More quotes from the participants can be found in the Supplementary Materials.

## Discussion

This paper aimed at appraising the EU position within the field of public health emergencies, taking experiences of the COVID-19 pandemic during the first wave, and it precedes the discussion on the EU 4 Health initiative (16). This study's main finding is that the limited mandate of the EU in health hinders most effective actions from preventing and tackling infectious disease outbreaks in, in this case, the COVID-19 pandemic. The results showed that this limitation significantly impacted the ECDC. ECDC has received many criticisms, but paradoxically EU MS did not give the agency greater capacity to operate more effectively. The European Commission has fulfilled its role of coordinating and supporting EU MS by facilitating networks and information exchange. However, EU intra- and inter-communications need further improvement. Although diverse EU instruments and mechanisms were found valid, their implementation needed to be faster and more efficient. The results indicate that underlying political challenges of EU decision-making regarding health emergencies hinder the aligned response. It was stated that the EU MS were ill prepared; the lack of legal competence concerning health for the EU institutions prevented the EU from enforcing binding guidelines. Thus, the EU response to the pandemic was not harmonized, and the pandemic revealed the individualistic approach and absence of solidarity among EU MS. In addition to this, the study elaborated on future EU pandemic strategies and actions. Interestingly, both EU institutions and EU MS experts came up with similar and clear recommendations regarding the ECDC, investment in resources, and the need for harmonized preparedness tools.

The EU's weak response to the current pandemic is to some extent explained by its limited legal mandate concerning public health. As stated in both the Treaty on the Functioning of the EU (TFEU) and the Charter of Fundamental Rights of the EU, the EU has no binding power regarding health. Since the EU MS have decided to maintain primary sovereignty on public health (granting only shared competence at best to the EU to assist the EU MS efforts concerning public health, particularly through Article 168 of the TFEU), the EU cannot enforce any guidelines regarding the organization and delivery of national health systems [Article 168 (7)]. Thus, a unified and centralized EU response for COVID-19 was legally not feasible (6). But the challenges of the crisis that has exposed the EU's weaknesses in public health preparedness and response provide also the opportunity for change. Brooks and Geyer argue that earlier health crises like SARS 2003, H1N1 2009 had triggered “incremental but important steps in the development and integration of EU health policy” (17). Before the crisis hit and under the Jean-Claude Juncker's Presidency health had slipped down the Commission's agenda resulting in speculations that the Health Directorate (DG SANTE) might even be discontinued as an independent directorate within the Commission's portfolio of directorates. But then in late 2019, with the new Presidency from Ursula von der Leyen, the health Directorate received a wider mission. The European Commission has with a 1.7 billion Euro health budget tripled the originally intended amount for the 2021–2027 period. This steep increase in funding will mainly cater to support overall pandemic response and health system strengthening for future infectious diseases outbreaks but will also provide the potential to further align and strengthening the EU health mandate overall.

Further, although the ECDC has performed risk assessments and shared transparent information since January 2020, its leadership role during this pandemic has been criticized by the participants in this study. The agency's actions were not visible enough to the public and national entities, and their recommendations were too vague. However, due to low political interdependence in public health at the EU level, the ECDC faces legal and functional restrictions (11). The involvement of the ECDC has been hindered by the EU MS' protectiveness of their competencies on public health. Increasing ECDC's capacities depends solely on the willingness of the EU MS to give up competencies and oversight to ECDC (7).

In addition to the ECDC, the EC has fulfilled its complementary role of coordinating EU MS to create a network and to facilitate the exchange of information by organizing COVID-19 special working groups, launching the Joint Procurement Mechanism (RescEU stockpiling), and through the work of the Health Security Committee, the Emergency Support Instrument and the Civil Protection Mechanism for the repatriation of EU citizens. Those health crisis instruments have supported the efforts of the unprepared EU MS, but they needed to be implemented faster and more efficiently. Political and doctrinal struggles and complex decision-making processes partially caused this delay at the beginning of the crisis (18): when Italy urged the activation of the RescEU, it was slowed by three EU MS, or when the self-styled “Frugal Four” (Netherlands, Austria, Denmark, and Sweden) opposed the EU recovery fund (18–20). Further, flaws in inter- and intra-communication have weakened the EU's position to be a strong actor during the first wave. While some DGs cooperate very well, the multi-sectoral structure required for Health in All Policies is not yet efficiently and adequately established (19, 21). This study showed how complicated the interaction between the ECDC, and other sectors was and how urgent it is to establish an EU Public Health Agency with the capacity to communicate with all parties: effective coordination and communication between governments, relevant agencies, and the EU are essential to tackle future crises.

Moreover, outbreak preparedness at all levels from regional, national, and EU level was the major weakness identified in this study. Investment and interest in health emergency preparedness at the national level have been neglected for several years, although experts and the WHO have repeatedly warned about future pandemics (18, 22). One reason that frequently appeared in the results was the belief that infectious diseases would break out mainly in developing countries and that the EU region was invulnerable due to its economic position in the world (23).

The observed limitations in preparedness are attributed to the insufficient implementation of the International Health Regulations and its monitoring, which were already pointed out during the H1N1 and Ebola outbreaks (24). Along with this, critical issues of transparency and accountability between EU MS and EU institutions in data sharing were identified in this study. Those problems were already observed during the Ebola outbreak (22). The structural issues in preparedness and response of the EU MS, such as their frail national surveillance system and infrastructure capacity, impacted the ability of the EU to ensure a collective response against the pandemic.

From a global perspective the EU's failures and shortcomings are not unique. In a recent contribution to *Foreign Affairs*, Ashish Jha points at the major shortcomings of global health governance that has been once more exposed by the COVID-19 pandemic and further exacerbated by member countries using the WHO as a battle ground for geopolitical competition (25). In an analysis of the COVID-19 response by countries that had participated in the 2016 Joint External Evaluation (JEE), a voluntary exercise to assess a country's level of preparedness to meet the obligations resulting from the IHR, Lee and Frieden found that countries could have scored high in the JEE and still be found performing poorly in the actual pandemic response due to the lack of responsible political leadership. The authors refer to the USA as a prime example of this failed leadership (26) but from a European perspective the lack of responsible political leadership—especially in the early phase—could also be found closer to home. Reflecting on Richard Horton's description of the current COVID-19 pandemic as a “syndemic” (27), Alami et al. (28) concluded in a recent study on the COVID-19 response in the province of Quebec, that one first lessoned learned would be to avoid a too narrow crisis management approach. Rather, they suggest to include also the dynamics and interactions resulting from inequity and vulnerabilities in society.

China counts among the examples of a comparatively fast and rather effective pandemic response. In 2003 the Chinese government had seen the lack of preparedness to respond to the SARS outbreak as an embarrassment and invested in the aftermath of the 2003 crisis in a comprehensive surveillance and response system including active case finding (29). With the current growing anti-China sentiment and the widespread perception of the comprehensive containment measures as being far too rigid and undemocratic the chance could be missed to learn from China's domestic successes (30). New Zealand and Australia, generally not being suspicious of antidemocratic governments, applied as well very rigid containment measures in combination with strict surveillance that showed good public health results.

In conclusion, the pandemic has revealed that EU MS' sovereignty in health was valued above the EU principle of solidarity. This was seen in the hoarding by EU MS of medical supplies, implementing different response strategies, and lengthy negotiations for developing the recovery funds and action plan (16–18). This was already observed during the H1N1 pandemic when EU MS prioritized their respective national interests and acted independently (7). Nonetheless, this pandemic once again showed and continues to show that individualistic national measures are still not functioning, and more cohesion and solidarity are needed to meet the realities of pandemics. The complex politics of public health governance at the EU level have led to a fragmentation affecting any effective pandemic responses. The ongoing pandemic has shed light on the fragility of the political and structural systems in Europe during public health emergencies. Lessons from past outbreaks were not learned, and warnings were not taken seriously. Still, hopefully, this COVID-19 pandemic will give fresh impetus to new and robust health reforms at the local, regional, national, and EU levels. The strengthening of EU health mandate by e.g., the joint procurement act will further engage the EU in the sensitive debate about a general expansion of the EU health policy mandate (17). The recent evaluation report on the global functioning of the International Health Regulations (31) has highlighted that the major deficiency was not with gaps in the International Health Regulations but with a lack of implementation and coherent translation into national laws and regulations supported by a collaborative and solidary attitude. The report concludes that “trust and transparency are the founding principles” for any effective and successful collaboration under the International Health Regulations (31). If—as our study shows—this trust and transparency is missing even among the 27 EU MS that have voluntarily joined the European Union as they share common culture, history and principal values, how can we expect that this is working on a global scale?

## Limitations

Our findings have to be viewed with caution and by considering some critical limitations. Our findings are based on a limited number of expert interviews covering the first wave of the pandemic in Europe. Expert opinions and views are not free from subjectivity and distortion. The number of participants (n=17) does not represent the EU's political organization and the small sample size is also a compromise of balancing coverage with contributing to the timely discussion.

## Recommendations

- The ECDC should get more capacity and funds to foster and implement International Health Regulations at the EU, national and regional levels and supervise their strict implementation.
- The shortcomings of the Covid-19 Pandemic need to be identified and analyzed for future crises to enhance preparedness in the EU.
- The EU legal framework should be revisited to allow audit implementation and allow a more coordinated and united EU response.
- The EU solidarity should extend into the more affluent EU MS supporting those EU MS that do not have (financial) capacity to develop such systems.
- The EU institutions should develop a common approach for tackling pandemics, such as standardized guidelines and training, enforced at the EU level and based on scientific evidence.
- At the EU MS level, global health security should be a critical item on their national agenda, working toward an improved and resilient coordinated national health system.
- Strengthening the local, regional and national surveillance system should be of high priority taking cross border surveillance within into account.
- Increase capacity, including equipment like intensive care beds, medical equipment, laboratory and analytical ability, and leadership skills in public health.
- New strategies should be developed for strengthening and enhancing cross-border emergency collaboration.

## Conclusion

The complex politics of public health at the EU level have led to the fragmentation of its governance for effective pandemic responses. This ongoing pandemic has shed light on the fragility of the political and structural systems in Europe in public health emergencies. Lessons from past outbreaks were not learned, and warnings were not taken seriously. Still, hopefully, this COVID-19 pandemic will give new impetus to new and robust health reforms at the local, regional, national, and EU levels.

## Data Availability Statement

The datasets presented in this article are not readily available because qualitative interview data is not made available due to privacy and data protection requirements and undertakings. Inquiries on the datasets should be directed to m.gontariuk@maastrichtuniversity.nl.

## Ethics Statement

The study received ethical clearance (FHML/GH_2018.076) from the Ethical Committee FHML-REC of Maastricht University, The Netherlands. The participants provided their written informed consent to participate in this study.

## Author Contributions

MG, TK, and EP conceptualized and designed the study. MG conducted the interviews, analyzed them, and wrote the original manuscript. EP, TK, and CR critically reviewed, commented, and revised the original draft. DT and LV reviewed the manuscript and added their legal expertise. All authors have contributed to the writing of the manuscript, they have read and approved the final version.

## Conflict of Interest

The authors declare that the research was conducted in the absence of any commercial or financial relationships that could be construed as a potential conflict of interest.

## Publisher's Note

All claims expressed in this article are solely those of the authors and do not necessarily represent those of their affiliated organizations, or those of the publisher, the editors and the reviewers. Any product that may be evaluated in this article, or claim that may be made by its manufacturer, is not guaranteed or endorsed by the publisher.
