# Supplementary material for: The European Union and Public Health Emergencies: Expert Opinions on the Management of the First Wave of the COVID-19 Pandemic and Suggestions for Future Emergencies
Source: Front Public Health. 2021 Aug 20;9:698995. doi: 10.3389/fpubh.2021.698995 (PMC8417533; doi:10.3389/fpubh.2021.698995)
Supplement: Supplementary file 1 [file Table_1.DOCX]

Supplementary Material

# Supplementary Data

**GUIDE FOR INTERVIEWS: EU SWOT during public health emergency- COVID 19**

1. ***General information***
   1. Could you please explain what are your current position and responsibilities (within the EU/health sector)?
2. ***Strength of EU in public health emergency situations like COVID-19***
   1. What is the strength of EU during the current COVID-19 public health emergency?
   2. What is the EU strength during COVID-19 in international/global health?
   3. In your opinion, was the EU public health institutions such as the ECDC effective?
3. ***Weaknesses of EU in public health emergency situations like COVID-19***
   1. What are the weaknesses of EU during the current COVID-19 public health emergency, if any?
   2. What is the EU weakness during COVID-19 in international/global health?
4. ***Opportunities of EU in public health emergency situations like COVID-19***
   1. What are the opportunities for the EU during the current COVID-19 public health emergency?
   2. Do you think the current crisis is an opportunity to reshape/reorganize the role of the EU in public health emergency? ECDC role?
   3. Do you think this is an opportunity for a new EU policy on public health emergencies and on global health?
5. ***Threats of EU in public health emergency situations like COVID-19***
   1. What are threats for the EU during the current COVID-19 public health emergency?
   2. What are the legal and political boundaries for the EU to manage public health emergencies?
   3. Do you think the EU is providing enough support for harmonizing public health emergency preparedness and responses?
   4. What EU agencies need to be enhanced to better manage public health emergencies like Covid-19?
6. ***Cooperation***

6.1. Do you think the EU is performing well in regard to cross border collaboration during COVID-19 / Meaning how much is left of cross-border collaboration when everyone turns to individual national strategies?

1. ***EU Role in Global Public Health***
   1. Do you see the EU’s role in Health and Public Health being strengthen or weakened in the future as a consequence of the Covid-19 crisis?
   2. What do you think in general about the EU role in the field of global/public health? (its importance)?
   3. What are lessons to be learned out of the current covid-19 crisis regarding the EU’s future approach and commitment to Global Public Health?
   4. What would be your vision for EU in regard to Global Health strategies?

# Supplementary Figures and Tables

**Table 2. Prominent themes, subthemes, and illustrative quotes.**

| **Themes** | **Subthemes** | **Illustrative Quotes** |
| --- | --- | --- |
| Experiences from EU institutions during the COVID-19 crisis | Collaboration | *“DG SANTE is a very, very important partner for us. And I dare to say that they feel the same way about us … So we have been working very closely together. We used over the recent months, every morning, during the course of the week, we had a meeting just to discuss the most recent development. … So, we do have a very close connection with them. And it has been functioning very well, I have to say that I have been quite pleased about the level of cooperation that we have with them.” (P14)* |
|  | Challenge | *“I've seen this in the past emergencies, in a way there is more to say your emergency is not my crisis, if you have an emergency, I mean, when I'm in crisis, I don't care about your emergency, in a way I need to manage my crisis, and that in a way goes beyond the principles of solidarity and global health, the global health principles are very much sustainable in peacetime. When the emergency strikes, all this, it's very fragile.” (P1)*  *“I would really encourage the member states to share their level of preparedness to us. I mean, I know that in some member states, there's a strong feeling that this is a kind of a national security issue. … And, you know, it's classified information and they are not in a position to share that with us. And it's certainly partially true, but I at the same time, I would believe that it would be very helpful if the member states would be willing to, at least in broad terms, explain how they are prepared for any incident that might take place”(P14)*  *“The systems which we had in place to be able to project support to the Congo or to Brazil or to the Middle East, or to China or wherever the problem was, are not really relevant when the problem is here. And I think that was a big discovery for many people. Because it was naturally assumed that everything, we had here was perfect. Therefore, we could properly deal with any problem that is just in reality, I think it's been shown that the countries were less than adequately prepared for this type of threat. And the many reasons for that because of course, it's difficult to invest in preparedness when there are calls on you to pay for new treatments for cancer patients or, you know, innovative medicines that need to be produced and so on. So, the attractiveness of investment in preparedness is often at a lower level politically than the investment in a new cancer ward”(P8)* |
| COVID-19 crisis experiences from some EU Member States | Coordination | *“I don't think they expected actually that virus will be spread so fast and they really we do not have one preventive action actually … European Union, it didn’t seem like a union actually in this situation”(P10)*  *“the strength of EU is being able to research on a much broader scale and easily share findings and data between member states and across borders and to provide support for countries within the EU that are most affected” (P18)* |
|  | Joint procurement | *“I think the EU didn't realize how to act, they were too slow to act because for example, the joint procurement mechanisms, … there were minimum problems in actually getting to that but also the mechanism that is there is too slow for this kind of activity” (P2)*  *“It is clearly obvious that the member states are not able to do it alone on their own, then the EU institutions can take over and push for joint regulations and procurements, etc.” (P12)*  *“The Commission has made a big effort, but also they have not addressed when you are on the field that you have to manage a pandemic, and you have to solve problems. The commission with actions like joint procurement, they don't really release very quickly the joint procurement, but now three months, four months later, the results are very small and so, every member state has solved the problem by themselves… I think the joint procurement is very clear but there were problem of personal equipment, problem of the Spanish government, French government, German government, not the problem of the Commission” (P15)* |
|  | ECDC | *“So, the ECDC, we are very keen to see them doing the surveillance, to have good benchmark of data between countries, … they are working on this they are doing quite the normative way of producing data over analysing. It's quite good, I would say. But if they start to produce propose recommendations for joint actions … but I feel the member states are not so keen for it and even the EU institutions, they said okay the agency is doing something but we don't want them to interfere with the way we deal with countries and we don't want them to go too far and say that some countries are not ready or, then there would be actions to be taken to say, okay, there are countries which are weak in the mechanism” (P12)*  *“I think ECDC has provided materials, but it's been quite invisible” (P2)*  *“I think the ECDC really showed that it's a totally irrelevant institution. It was not there when it was when it was needed most. It really did not function, did not provide guidance or recommendation is not provided initially, certainly initially … It prove that it's basically some organization which is very, very much a political type of organization with very little public health or evidence-based policies drive. It probably picked up after a few months realizing that it actually does need to do something in order for it to justify its existence. So then started to look into the research and picking up evidence from different countries and bringing evidence of different countries and data systems together. As of late, it's started becoming more useful than before, but in truth, it responded very poorly.With or without it, it certainly initially made absolutely no difference” (P7)* |
|  | Preparedness | *“There's a significant EU budget going towards communicable diseases. … However, I think a lot of it was focused on Africa, Asia, etc. And I think it shows that we were not entirely prepared … I think that we are not used anymore that we could die of an infectious disease. And I think that is translated also to the level where we designed the subsidies at the EU level” (P17)*  *“We in Europe think that we know everything. We are prepared for everything, but we show actually our weakness in this crisis.”*  *“There's been this awkward situation where the pandemic preparedness had been done to my mind just solely on the basis on the ground of influenza pandemic. So, the people have been kind of prepared, but they've not realized that this particular kind of a pathogen is not influenza, or it's not behaving in the same way as influenza” (P2)*  *“Europe has been putting the focus in the last 15 years in health care systems, and they have forgotten the communicable diseases thinking that communicable disease is something outside the borders of European region and the same with the European Union.” (P15)* |
|  | Political barriers | *“We have systems and they have been used and they are working. So I think that's a good thing. And then of course, we do have tools. We do have teleconferences and we do have interaction with each other on a technical level, but I think the technical level may not be the biggest problem. I think the biggest problem is the political level.” (P11)*  *“politically very difficult, if not impossible to make the step because if you give competencies to the EU you lose competencies and the member states” (P3)* |
| Future actions for public health emergencies at EU level | EU institutions’ suggestions for improvements | *“I don't think that the regulatory power would increase the capacity of the ECDC to control an outbreak is not that immediate the main mechanisms, probably what would be needed is to get to more capacity to interact with a political entities in Europe”(P1)*  *“we need to look in the context of reviewing the ECDC mandate and the cross-border health care decision to see what improvements we could propose, without necessarily going as far as a treaty change, which would, of course, be trickier and more complicated” (P8)* |
|  | Member states’ suggestions for improvements | *“That is coherent that you get the same measures and that was also confusing in this crisis for member states and for the population, that every country has different rules” (P9)*  *“I think what COVID-19 made really clear is that you can't think global health as something for the others, this is about a local health and how we deal with it. In that sense, I think within the EU it is about rethinking about the EU regulations and accommodating the broader needs of societies. … The EU need to recognize that the EU social policy model has to be strong and it has to have a kind of role for public services because I think for too long they've been digging out the Public Services and Public Procurement, it's been kind of commercialized and emphasizing too much on internal market” (P2)* |
|  | Vision/opportunity | *“I've seen this past crisis during the crisis and just after the crisis, everybody there's no reset, lessons learned exercises. So yes, we have done that and it is quite important but then the main lessons that remain that it's learned is that nothing is learned. So we've seen this with Ebola, we see H1N1 pandemic, even before and with Zika. All this, you know, there's always a lot of initiatives that are just after to learn, then that we don't learn very much that is so in this case something should be learned from this is that this principles should be also very much based on public health” (P1)*  *“I think our systems are incremental in nature. And they built on experience from different crises. So every time we've had a crisis, the system has been improved and strengthened. It's unfortunate that things work that way, because it would be nice to have a perfect system which would answer any crisis but I think it's only when you have the crisis, that you discovered there are gaps”(P8)*  *“I hope so, there are signs that it will because the you know the new EU for health program was suppose I don't know if it's still true I would be very attentive to it - And I hope the world has understood because I'm not sure about it when I see what's going on in Europe. Everybody wants to forget. Let's forget about it. Let's forget totally. We will have surprises in the coming months because it's not finished” (P12)*  *“I think that these crisis are always an opportunity and the bigger, the bigger the opportunity, because, of course, your attention is on this now, we definitely want the case to get stronger in case it happens again, but a window of attention won't last forever. So the question of how much you can capitalize on that attention, you're trying to strengthen your preparedness to be ahead of next crisis”(P16)* |
